# Study on Leigh syndrome caused by *SURF1* gene mutations and its mechanisms

**DOI:** 10.3389/fneur.2026.1793054

**Published:** 2026-04-02

**Authors:** Chunmei Wang, Longlong Lin, Yuanfeng Zhang, Simei Wang, Xiaona Luo, Xuqin Chen

**Affiliations:** Department of Neurology, Shanghai Children’s Hospital, School of Medicine, Shanghai Jiao Tong University, Shanghai, China

**Keywords:** Chinese children, Leigh syndrome, mitochondrial DNA depletion, Shy1 domain, splice-site variant

## Abstract

Leigh syndrome (LS) is a prevalent mitochondrial encephalomyopathy in childhood, triggered by mutations in mitochondrial DNA (mtDNA) or nuclear DNA (nDNA). The protein encoded by the *SURF1* gene localizes to the inner mitochondrial membrane and is involved in the biosynthesis of the cytochrome c oxidase (COX) complex. We enrolled 5 children harboring *SURF1* gene variants whose clinical manifestations were highly consistent with LS. The clinical characteristics and potential pathogenic mechanisms of the disease were elucidated by systematic analysis of their clinical data. Among the 5 patients, 4 were female and 1 was male, with ages ranging from 13 months to 2 years and 7 months. Next-generation sequencing (NGS) results revealed 6 variant sites in the SURF1 gene among the 5 patients, of which 2 were known variants and 4 were unreported novel variants, namely c.314-317delTGCC (p.L105Qfs*7), c.588+1_588+3delGTA (splicing), c.655G>T (p.Glu219), and c.515+3G>C. Reverse transcription-quantitative polymerase chain reaction (RT-qPCR) was performed on the peripheral blood of 4 patients, and the results demonstrated that the messenger RNA (mRNA) expression level of the *SURF1* gene was significantly lower than that in their parents. Using 10 healthy children as controls, we analyzed the ratios of mitochondria-related NADH–ubiquinone oxidoreductase core subunit 1 (ND1), Cytochrome c oxidase subunit I (COX1), Cytochrome c oxidase subunit II (COX2), NADH–ubiquinone oxidoreductase chain 4 (ND4), Glyceraldehyde-3-phosphate dehydrogenase (GAPDH) was used as a nuclear reference gene. Mitochondrial DNA content was determined by measuring the ND1/GAPDH ratio using RT-qPCR, and further verified with COX1, COX2, and ND4. These ratios were all significantly decreased, indicating reduced mitochondrial DNA (mtDNA) copy number/mtDNA depletion. Iterative Threading ASSEmbly Refinement (I-TASSER)-based three-dimensional (3D) structural analysis indicated that all 6 variant sites induced alterations in the spatial structure of the SURF1 protein. The SURF1 protein is a hydrophilic protein, protein hydrophobicity and stability analyses showed that the 4 unreported novel variants could reduce the hydrophilicity, increase the hydrophobicity, and decrease the structural stability of the protein. The *Saccharomyces cerevisiae* Homolog of Yeast 1 (Shy1) domain serves as the key structural basis for SURF1 to exert its mitochondrial functions. We found that all 6 variant sites in the *SURF1* gene were located within the Shy1 domain.

## 1 Introduction

Leigh syndrome (LS, OMIM #256000), also referred to as subacute necrotizing encephalomyelopathy, is a common pediatric mitochondrial encephalomyopathy caused by mutations in mtDNA or nuclear DNA (nDNA) (1). This disease arises from impaired oxidative phosphorylation of the mitochondrial respiratory chain, which in turn leads to degenerative damage to the central nervous system, with a complex underlying etiology. To date, a variety of enzyme deficiencies have been identified as causative factors of LS, among which deficiency of mitochondrial respiratory chain complex IV (i.e., COX) is the most prevalent, inherited in an autosomal recessive manner (2). Notably, defects in the *SURF1* gene (MIM #185620) constitute the leading pathogenic factor in COX-related LS (3).

To date, a total of 814 variants in the *SURF1* gene have been reported, including 23 nonsense and 224 missense mutations. Most missense mutations are located in evolutionarily conserved regions, and 90 variants can cause LS and are mainly distributed within the conserved Shy1 domain of the SURF1 protein. Among the 5 patients enrolled in this study, 4 were female and 1 was male, with a total of 6 *SURF1* gene variant sites identified. Of these 6 variant sites, 4 have not been previously described in the literature databases, namely c.314-317delTGCC (p.L105Qfs*7), c.588+1_588+3delGTA (splicing), c.655G>T (p.Glu219*), and c.515+3G>C.

Reverse transcription-quantitative polymerase chain reaction (RT-qPCR) was performed on peripheral blood samples from 4 patients, revealing that their mRNA expression levels were significantly lower than those of their parents. Furthermore, 10 healthy children were recruited as controls to analyze the ND1/GAPDH ratio and the expression level changes of COX1, COX2, and ND4 in the peripheral blood of 3 patients. These ratios were all significantly decreased, indicating reduced mitochondrial DNA (mtDNA) copy number/mtDNA depletion, suggesting decreased SURF1 protein expression and insufficient mitochondrial DNA (mtDNA) content. I-TASSER-based three-dimensional (3D) structural analysis demonstrated that all 6 variant sites induced structural alterations in the SURF1 protein. Wild-type and all mutant protein sequences were uploaded to the SWISS-MODEL server, and alignment between wild-type and mutant proteins using PyMOL software revealed distinct differences in protein conformation. Protein hydrophobicity and stability analyses showed that the 4 variants not previously reported in literature databases could induce amino acid changes, leading to decreased protein hydrophilicity, increased hydrophobicity, and reduced structural stability. Notably, c.515+3G>C is an intronic mutation; RT-qPCR and intron sequencing analyses confirmed that this mutation caused abnormal splicing of the *SURF1* gene, resulting in exon 5 deletion. Consequently, the mRNA expression level was significantly lower than that in the patients’ parents and the normal control group, accompanied by altered protein structure and reduced structural stability, which may impair protein function. The present study aimed to enrich the *SURF1* gene variant spectrum by analyzing the clinical characteristics and gene variant sites of the aforementioned cases.

## 2 Materials and methods

This study was approved by the ethics Committee of Shanghai Children’s hospital (2019R07-F03). Informed consent to use blood samples for genetic analysis was obtained from the patient’s parents. Written informed consent was obtained from the parents of the children. Written informed consent was obtained from the [individual(s) AND/OR minor(s)’ legal guardian/next of kin] for the publication of any potentially identifiable images or data included in this article.

### 2.1 Next-generation sequencing (NGS)

Venous blood samples were collected from patients and their parents, which were then subjected to NGS analysis. Genomic DNA was fragmented using a Covaris ultrasonic disruptor and hybridized with the Roche NimbleGen 2.0 probe capture array to generate an exon-enriched DNA library. Library pre-capture was performed using the Illumina TruSeq DNA Sample Preparation Kit, followed by amplification via LCM-PCR. The Agilent DNA 1000 chip was used to assess library concentration, fragment size distribution, and quality, while quantitative polymerase chain reaction (qPCR) was employed to verify library enrichment efficiency and quality. Subsequently, the library was sequenced on the Illumina HiSeq 2500 platform, and base calling and raw data generation were conducted using the bcl2fastq software. Raw data with a quality score ≥20 (Q20) were filtered for subsequent genotype analysis. The Burrows-Wheeler Alignment Tool (BWA, version 0.7.15) was utilized to align sequencing reads to the human reference genome hg19 (UCSC Genome Browser). Rare variants (minor allele frequency [MAF] < 0.05) were filtered based on variant frequencies retrieved from the Exome Aggregation Consortium (ExAC) database. The pathogenicity of identified variants was predicted in accordance with the variant classification guidelines established by the American College of Medical Genetics and Genomics (ACMG). Variants detected by whole-exome sequencing were further validated by polymerase chain reaction (PCR) and Sanger sequencing.

### 2.2 Quantitative real-time polymerase chain reaction (qPCR) analysis of SURF1 gene expression and mitochondrial DNA levels

Total RNA was extracted from patients and their parents using the Qiagen RNA Preparation Kit, and complementary DNA (cDNA) was synthesized by reverse transcription using the PrimeScript™ Strand cDNA Synthesis Kit/RT Master Mix (TAKARA). Specific primers and dual-labeled probes for the SURF1 gene were designed as follows: forward primer 5′-GGTCGGAAGTGGAAGCTGAA-3′ and reverse primer 5′-AGAGTCCATACCAGGTCACGA-3′. The PCR reaction conditions were set as follows: pre-denaturation at 95.0 °C for 3 min, followed by 35 cycles of denaturation at 95.0 °C for 30 s and annealing/extension at 60.0 °C for 45 s. Three technical replicates were set for each sample, and the data were expressed as the mean ± standard error of the mean (SEM). Statistical analysis was performed using the 2^−ΔΔCt^ method. All detections were repeated three times, and the results were expressed as the mean ± standard deviation (SD). Intron sequencing was commissioned to Shanghai Sain Biotechnology Co., Ltd.

### 2.3 Structural modeling of SURF1 protein variants

The I-TASSER Suite analysis pipeline consists of four core steps: template sequence screening, iterative structural assembly simulation, model screening and optimization, and structure-based functional annotation. The accessible URL of this server is http://zhanglab.ccmb.med.umich.edu/I-TASSER.

The per-residue Local Distance Difference Test (pLDDT) scores indicate the reliability of the three-dimensional structure prediction for the SURF1 protein based on the AlphaFold model. Regions shown in blue represent parts of the structure where the prediction is highly reliable, whereas regions in red indicate areas that may be more flexible or have lower prediction accuracy. Gray part: Clearly indicates sequence changes and truncation regions caused by mutations. The other colored regions (blue, green, yellow, and red) represent the pLDDT scores from the AlphaFold model, indicating the predictive reliability of different protein segments. Blue: Very high confidence (pLDDT > 90); Green/Light Blue: High confidence (70 < pLDDT < 90); Yellow: Low confidence (50 < pLDDT < 70); Red/Orange: Very low confidence (pLDDT < 50), often corresponding to unstructured or disordered regions (4, 5).

Wild-type and all mutant protein sequences were uploaded to the SWISS-MODEL server, and sequence alignments between the wild-type and mutant proteins were performed using PyMOL software. The wild-type protein was labeled in green and the mutant proteins in red, with distinct conformational differences between them clearly visualized in the generated images.

### 2.4 Analysis of protein hydrophobicity and stability

The hydrophilicity index of each amino acid in the SURF1 protein was calculated using the Hphob./Kyte & Doolittle method in the ProtScale software.^1^ The grand average of hydropathicity (GRAVY) was used to evaluate the overall hydrophobicity or hydrophilicity of the protein: proteins with GRAVY < 0 were defined as hydrophilic, while those with GRAVY > 0 were defined as hydrophobic. Protein stability was analyzed using the ProtParam tool^2^ and expressed as the instability index, which ranges from 0 to 100; a higher value indicates lower protein stability.

## 3 Results

### 3.1 Clinical data

All 5 patients with *SURF1* gene variants exhibited characteristic clinical manifestations and genetic alterations (Table 1), as detailed below:

**Table 1:** Clinical features of five patients carrying *SURF1* variants.

Data	Case 1	Case 2	Case 3	Case 4	Case 5
Sex	Male	Female	Female	Female	Female
Age at examination	18 months	3 years old	21 months	11 months	18 months
Age at onset	13 months	16 months	15 months	No	18 months
Family history	No	No	No	No	No
Parental consanguinity	No	No	No	No	No
Perinatal risk factors	No	No	Her mother had a respiratory infection during pregnancy	No	No
Dystonia and its nature	Short stature, hirsutism, and inability to walk independently.	Short stature, hirsutism, bilateral esotropia, and nystagmus, bilateral esotropia (more prominent in the left eye) accompanied by nystagmus.	Short stature, hirsutism, and decreased muscle strength and tone	Short stature and hirsutism, with normal muscle strength and tone	Short stature and hirsutism, with normal muscle strength and slightly decreased muscle tone.
Blood lactate	4.2 mmol/L	3.9 mmol/L	5.5 mmol/L	3.5 mmol/L	2.9 mmol/L
cMRI	Abnormal signals in the dorsal thalamus, brainstem, junction of the medulla oblongata and pons, and cerebral peduncles	Multiple abnormal signals in the dentate nuclei of bilateral cerebellar hemispheres, the basis pontis, and the right basal ganglia region.	Multiple abnormal signal intensities in the bilateral basal ganglia	No abnormalities	Abnormal signal intensities in the brainstem and bilateral thalami, as well as fullness of the bilateral lateral ventricles
Gene mutation	c.314-317delTGCC (p.L105Qfs*7) and c.588 + 1_588 + 3delGTA (splicing) in SURF1	c.754-755del (p.Ser252Lisfs*39) and c.515+3G>C in SURF1	c.792_793del/p.Arg264Serfs*27 and c.655G>T/p.Glu219* in SURF1	c.792_793del/p.Arg264Serfs*27 and c.655G>T/p.Glu219*in SURF1	c.754-755del (p.Ser252Lisfs*39) in SURF1

Case 1: A 18-month-old male presented with delayed motor development compared with peers. He was diagnosed with developmental delay at 13 months of age in an outside hospital. The patient was the first child of a non-consanguineous family (G1P1), born full term via spontaneous vaginal delivery. His mother had an uneventful pregnancy, and there was no history of perinatal hypoxia or asphyxia. His parents were healthy, and had no parental consanguinity, with no family history of mitochondrial disease. Physical examination revealed short stature, hirsutism, and inability to walk independently. Auxiliary examinations showed elevated blood lactate at 4.2 mmol/L (normal range: 0.1–2.7 mmol/L). Cranial MRI demonstrated symmetric abnormal signals in the dorsal thalamus, brainstem, junction of the medulla oblongata and pons, and cerebral peduncles. Genetic analysis identified two heterozygous variants in the SURF1 gene: c.314-317delTGCC (p.L105Qfs*7) and c.588+1_588+3delGTA (splicing), both of which were unreported in the literature. The c.314-317delTGCC variant causes a frameshift mutation (p.L105Qfs*7) and was classified as likely pathogenic according to the ACMG guidelines. This variant has not been reported in public databases, and no pathogenicity assessment was available in the ClinVar database. Family segregation analysis confirmed that the patient inherited this variant from his heterozygous father, while his mother did not carry this variant. The c.588+1_588+3delGTA variant leads to abnormal splicing and was also classified as likely pathogenic according to the ACMG criteria. This variant was absent from public databases and ClinVar. Family validation showed that the patient inherited this variant from his heterozygous mother, while his father did not carry this variant. The patient died at the age of 3 years due to severe pneumonia and respiratory failure.

Case 2: A 3-year-old female presented with delayed motor development. She could stand alone unsteadily at 16 months of age and walk independently at 21 months of age. At 3 years old, she developed bilateral esotropia (more prominent in the left eye) accompanied by nystagmus. The patient was the second child (G2P2), born full term via spontaneous vaginal delivery. Her mother had an uneventful pregnancy, and there was no history of perinatal hypoxia or asphyxia. Her elder sister (G1P1) was healthy at 6 years old. Her parents were healthy, and had no parental consanguinity, with no family history of mitochondrial disease. Physical examination revealed short stature, hirsutism, bilateral esotropia, and nystagmus. Auxiliary examinations showed elevated blood lactate at 3.9 mmol/L. Echocardiography indicated atrial septal defect. Abdominal B-ultrasound showed left renal agenesis and compensatory enlargement of the right kidney. Cranial MRI demonstrated multiple abnormal signals in the dentate nuclei of bilateral cerebellar hemispheres, the basis pontis, and the right basal ganglia region. Genetic analysis identified two variants: c.754-755del (p.Ser252Lisfs*39) and c.515+3G>C, of which c.515+3G>C was previously unreported in the literature. Family segregation analysis confirmed that the patient inherited the c.754-755del (p.Ser252Lisfs*39) variant from her heterozygous mother, while her father did not carry this variant. For the c.515+3G>C variant, the patient inherited it from her heterozygous father, while her mother did not carry this variant. The patient died at the age of 3 years and 8 months due to severe pneumonia and respiratory failure.

Case 3: A 21-month-old female presented with delayed motor development. She could walk with support at 15 months of age but was unable to walk independently. Motor regression occurred at 18 months of age, with a steppage gait during assisted walking and generalized tremors when walking with assistance or squatting to pick up objects. The patient was the first child (G1P1), born full term via spontaneous vaginal delivery. Her mother had a respiratory infection during pregnancy, and there was no history of perinatal hypoxia or asphyxia. Her younger sister (G2P2) carried the same gene mutations. Her parents were healthy, and had no parental consanguinity. Physical examination revealed short stature, hirsutism, and decreased muscle strength and tone. Auxiliary examinations showed elevated blood lactate at 5.5 mmol/L. Cranial MRI demonstrated multiple abnormal signal intensities in the bilateral basal ganglia, dentate nuclei, central semiovale, and brainstem. Two heterozygous mutations in the *SURF1* gene were identified in genomic DNA extracted from the patient’s peripheral blood: c.792_793del/p.Arg264Serfs*27 and c.655G>T/p.Glu219*. Family segregation analysis confirmed that the patient inherited thec.792_793del/p.Arg264Serfs*27 variant from her heterozygous father, while her mother did not carry this variant. For the c.655G>T/p.Glu219* variant, the patient inherited it from her heterozygous mother, while her father did not carry this variant.

Case 4: A 11-month-old female, the younger sister of Case 3, The patient was the second child (G2P2), born full term via spontaneous vaginal delivery. Her mother had an uneventful pregnancy, and there was no history of perinatal hypoxia or asphyxia. Her parents were healthy and had no parental consanguinity. Physical examination revealed short stature and hirsutism, with normal muscle strength and tone. Auxiliary examinations showed a blood lactate level of 3.5 mmol/L. Cranial MRI demonstrated a small amount of abnormal signal intensity adjacent to the posterior horns of bilateral lateral ventricles, consistent with incomplete myelination, and slightly widened extracerebral spaces in the bilateral temporal regions. Because of the abnormal genetic findings in her elder sister (Case 3), the patient underwent familial validation at 11 months of age and was found to carry the same variant sites. She subsequently received rehabilitation therapy.

Case 5: A 18-month-old female presented with the ability to walk with support but inability to walk independently at 18 months of age. The patient was the first child (G1P1), born full term via spontaneous vaginal delivery. Her mother had an uneventful pregnancy, and there was no history of perinatal hypoxia or asphyxia. Her parents were healthy, and had no parental consanguinity, with no family history of mitochondrial disease. Physical examination revealed short stature and hirsutism, with normal muscle strength and slightly decreased muscle tone. Auxiliary examinations showed a blood lactate level of 2.9 mmol/L. Cranial MRI demonstrated abnormal signal intensities in the brainstem and bilateral thalami, as well as fullness of the bilateral lateral ventricles. The genetic variant identified was c.754-755del (p.Ser252Lisfs*39). Parental genetic testing confirmed that this variant was inherited from her father.

### 3.2 Detection of variant sites

A total of six *SURF1* gene variants were identified in this study.

Case 1 carried two variants: c.314-317delTGCC (p.L105Qfs*7) (frameshift mutation) and c.588+1_588+3delGTA (splicing).

Case 2 carried two variants: c.754-755del (p.Ser252Leufs*39) (frameshift mutation) and c.515+3G>C.

Case 3 carried two variants: c.792_793del (p.Arg264Serfs*27) (frameshift mutation) and c.655G>T (p.Glu219) (nonsense mutation).

Case 4 carried the same two variants as: c.792_793del (p.Arg264Serfs*27) (frameshift mutation) and c.655G>T (p.Glu219*) (nonsense mutation).

Case 5 carried one variant: c.754-755del (p.Ser252Leufs*39) (frameshift mutation).

Among them, the variants c.314-317del TGCC (p.L105Qfs*7), c.588+1_588+3delGTA (splicing), c.655G>T/p.Glu219*, and c.515+3G>C were novel variants with no previous literature reports. Analysis using public databases including gnomAD, Exome Aggregation Consortium (ExAC), and 1,000 Genomes revealed that none of these variants were recorded in the general population databases.

### 3.3 Analysis of *SURF1* gene expression and mitochondrial DNA levels

**Figure 1:**
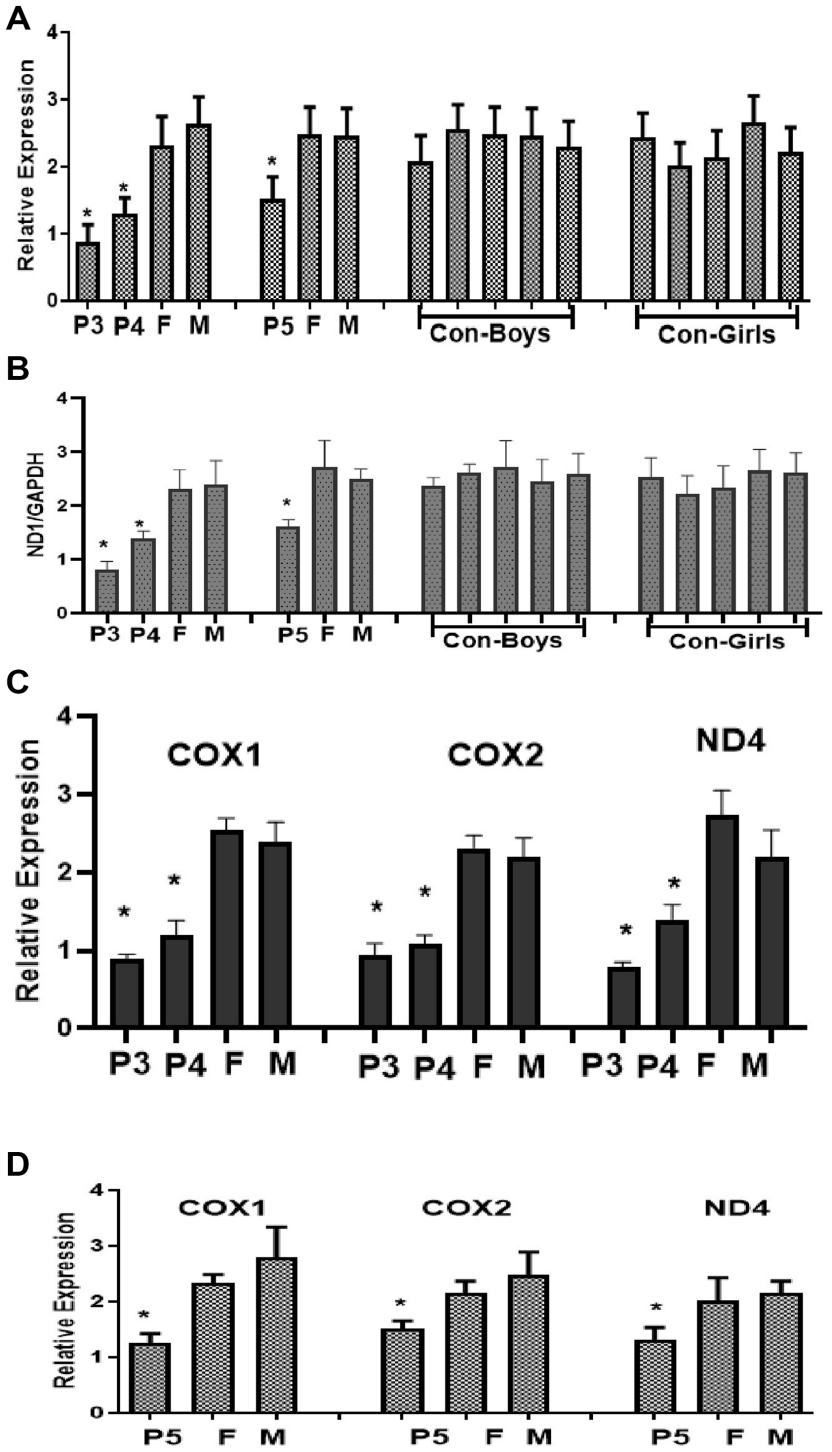
Analysis of *SURF1* gene expression and mitochondrial DNA levels in Case 3–5. **(A)** The expression level of the SURF1 gene in probands 3–5 (P3P5) was significantly lower than those in their parents and normal controls. **(B)** The expression level of the ND1/GAPDH ratios in probands 3–5 (P3P5) was significantly lower than those in their parents and normal controls. **(C)** The expression level of the COX1, COX2, and ND4 in probands 3–4 (P3, P4) was significantly lower than those in their parents and normal controls. **(D)** The expression level of the COX1, COX2, and ND4 in probands 5 (P5) was significantly lower than those in their parents and normal controls.

**Figure 2:**
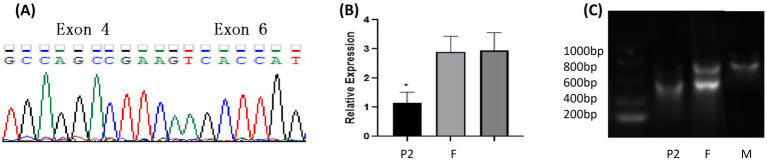
Genetic analysis and expression of *SURF1* gene variants in Case 2. **(A)** The probands 2 (P2) intronic variant c.515 + 3G > C caused abnormal splicing of the *SURF1* gene, leading to the deletion of exon 5. **(B)** The expression level of the SURF1 gene in probands 2 (P2) was significantly lower than those in their parents. **(C)** PCR results of cDNA from the nuclear family of probands 2 (P2), the amplicon in the proband was shorter than the corresponding bands in the parents.

To investigate whether nonsense mutations cause disease by affecting protein synthesis or abundance, RT-qPCR analysis was performed in four pedigrees following informed consent from the patients’ families, qPCR analysis. The expression level of the *SURF1* gene in probands 2–5 (P2, P3, P4, P5) was significantly lower than those in their parents and normal controls (Figures 1A, 2B,C) (the parents of Case 1 declined to provide blood samples and were thus excluded from the analysis).

Leigh syndrome caused by *SURF1* gene abnormalities is characterized by an early onset, with the majority of affected children succumbing before 10 years of age. To evaluate alterations in mtDNA content, 10 normal children aged 1.5–10 years (5 boys and 5 girls) were enrolled as the control group. The conserved mitochondrial gene ND1 was selected to represent mtDNA, while the conserved nuclear geneGAPDH was used as a reference for nuclear genomic DNA. The ND1/GAPDH ratio from Case 3 to Case 5 was employed to reflect the relative content of mtDNA to nuclear DNA, where a decreased ratio indicates reduced mtDNA content. Additionally, the expression levels of COX1, COX2, and ND4 (mitochondrial genes) were analyzed. The expression level of the ND1/GAPDH ratios in probands 3–5 (P3, P4, P5) was significantly lower than those in their parents and normal controls (Figure 1B). Consistently, the expression level of the COX1, COX2, and ND4 in probands 3–4 (P3, P4, P5) was significantly lower than those in their parents and normal controls. The expression level of the COX1, COX2, and ND4 in probands 5 (P5) was significantly lower than those in their parents and normal controls (Figures 1C,D), suggesting decreased SURF1 protein expression and insufficient mitochondrial DNA (mtDNA) content, and lead to insufficient mtDNA content in affected patients.

### 3.4 Protein structure prediction mutations via the I-TASSER server, with cross-validation by SWISS-MODEL and PyMOL

**Figure 3:**
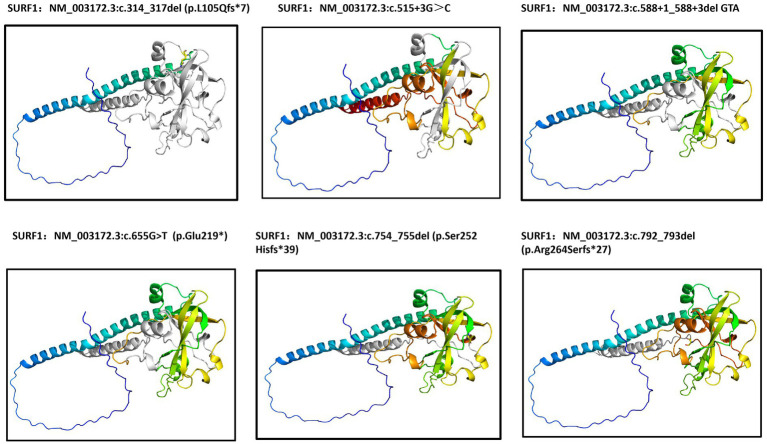
The structures of wild-type and six mutant SURF1 were modeled using I-TASSER. Protein structure prediction analysis demonstrated that the mutations caused protein truncation, leading to structural impairment. Gray part: Clearly indicates sequence changes and truncation regions caused by mutations. Regions shown in blue represent parts of the structure where the prediction is highly reliable, whereas regions in red indicate areas that may be more flexible or have lower prediction accuracy.

I-TASSER software was used to predict the amino acid changes caused by five *SURF1* gene variants, including c.754_755del (p.Ser252Hisfs*39), c.792_793del (p.Arg264Serfs*27), c.314-317delTGCC (p.L105Qfs*7), c.655G>T (p.Glu219*), and c.588+1_588+3delGTA (splicing). Compared with the wild-type SURF1 protein, all the aforementioned variant sites resulted in alterations in the three-dimensional (3D) structure of the protein, suggesting that its inherent stable structure was disrupted (Figure 3).

The intronic variant c.515+3G>C caused abnormal splicing of the *SURF1* gene, leading to the deletion of exon 5. *In vivo* experiments confirmed that the variant product was NM_003172.3: c.324_515del (p.Asp108_Gly172delinsGlu) (Figure 2A). Three-dimensional (3D) protein structure prediction showed that this variant resulted in truncation of the amino acid sequence, which in turn altered the 3D structure of the protein (Figure 3).

**Figure 4:**
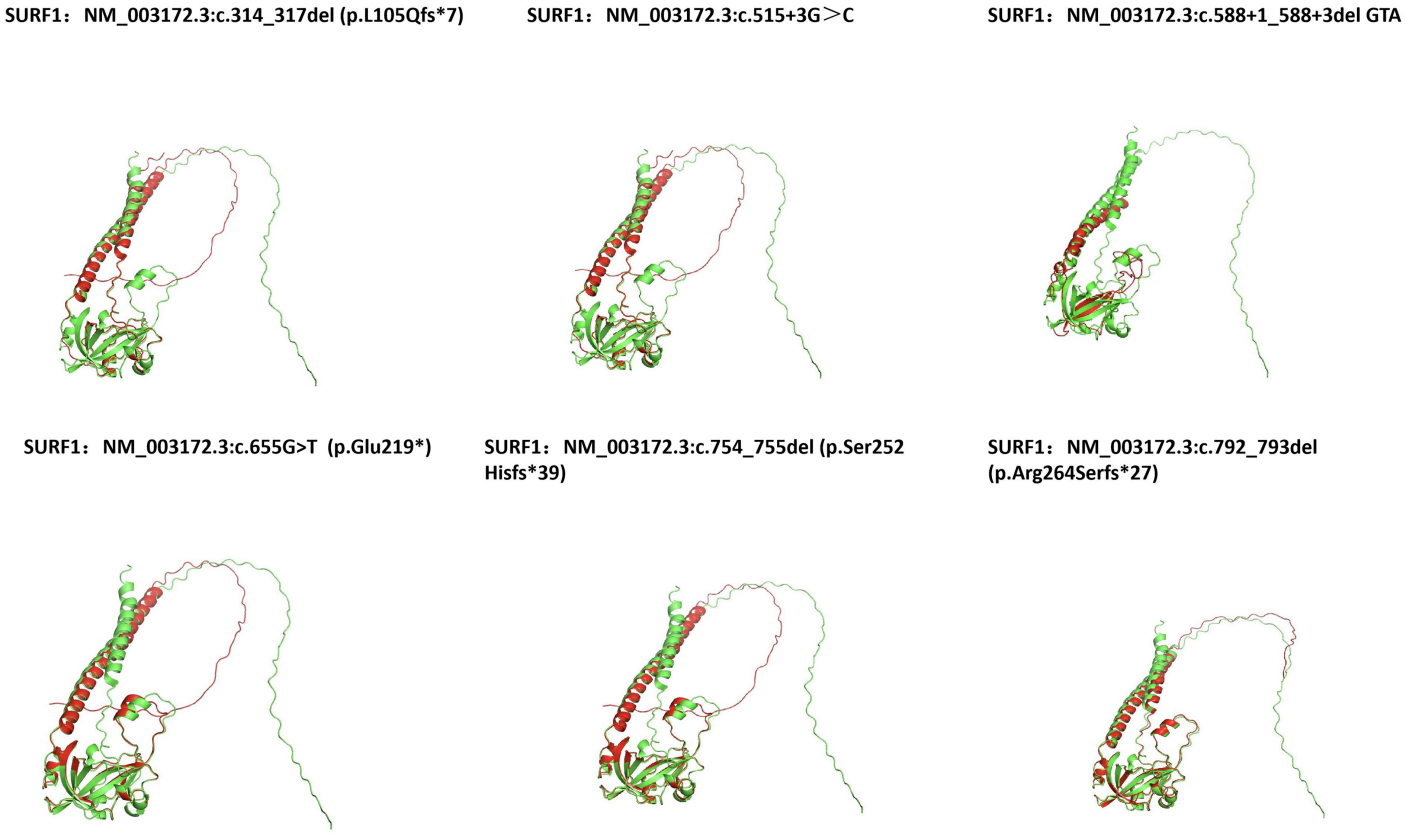
Protein structure prediction mutations with cross-validation by SWISS-MODEL and PyMOL. The wild-type protein was labeled in green and the mutant proteins in red, with distinct conformational differences between them clearly visualized in the generated images.

Additionally, wild-type and all mutant protein sequences were uploaded to the SWISS-MODEL server, and alignments between the wild-type and mutant proteins were performed using PyMOL software, which revealed differences in protein conformation (Figure 4).

### 3.5 Analysis of protein hydrophobicity and stability

**Figure 5:**
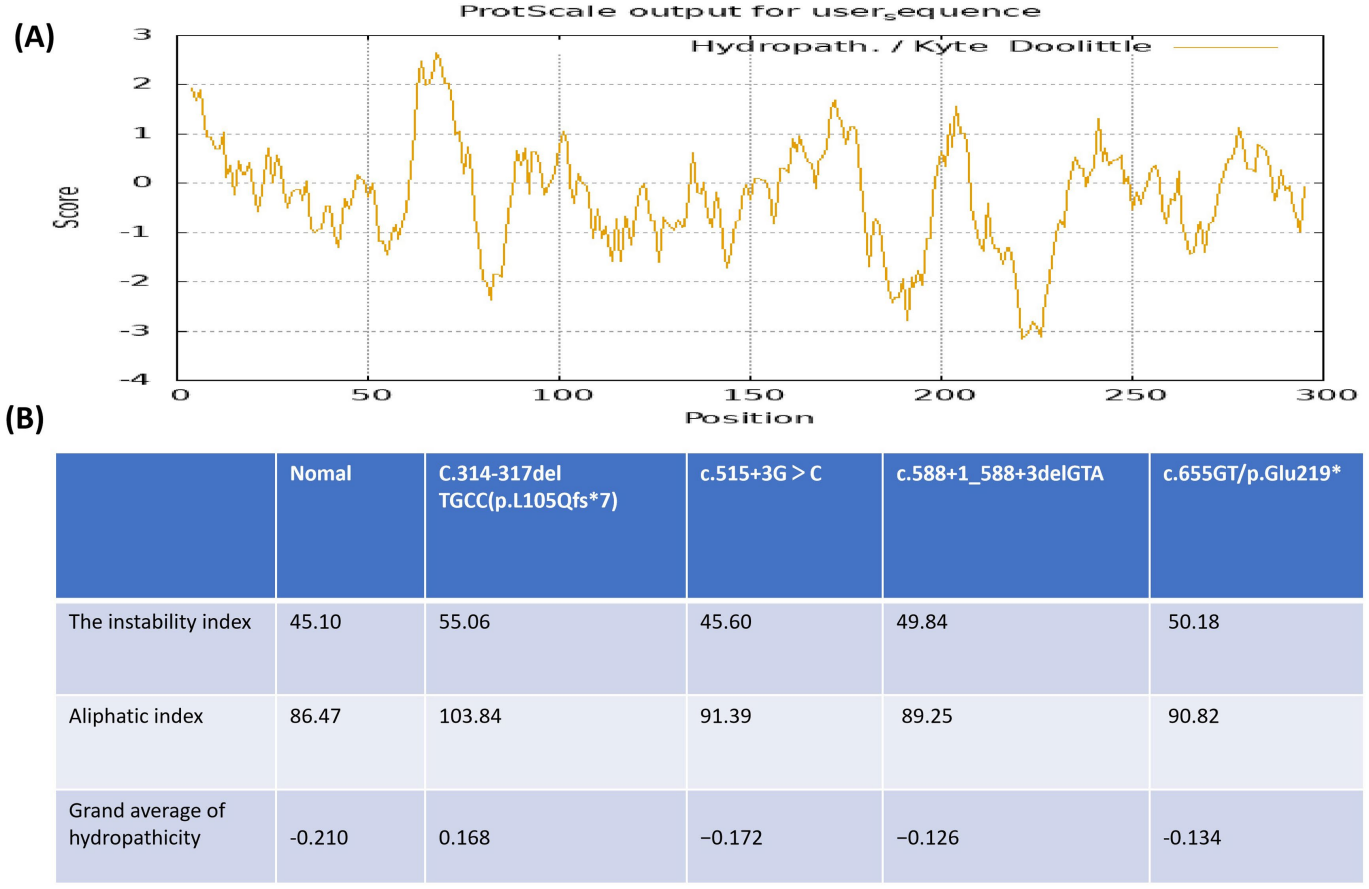
Analysis of protein hydrophobicity and stability. **(A)** The predicted hydropathicity index of each amino acid residue in the wild-type SURF1 protein. **(B)** The predicted hydropathicity profile of the mutant SURF1 protein harboring each of the four novel mutations.

Analysis using ProtScale software showed that the hydrophilic regions of the SURF1 protein were located at amino acid positions 29–47, 53–61, 79–89, 104–158, 181–199, 209–234, and 288–296 (Figure 5A). Prediction by ProtParam software revealed that the wild-type SURF1 protein had a grand average of hydropathicity (GRAVY) of −0.210 (indicating hydrophilicity), an instability index (II) of 45.10, and an aliphatic index of 86.47. A more negative GRAVY value indicates higher hydrophilicity and greater protein structural stability, whereas a higher aliphatic index is associated with higher hydrophobicity and lower protein structural stability. Additionally, a higher instability index reflects lower protein structural stability. The SURF1 protein is a hydrophilic protein; therefore, any abnormality in its structure that reduces hydrophilicity and increases hydrophobicity will lead to structural instability.

The protein characteristics corresponding to the four novel *SURF1* gene variants are as follows (Figure 5B):

c.314-317delTGCC (p.L105Qfs*7): grand average of hydropathicity (GRAVY) = 0.168 (hydrophobic); instability index (II) = 55.06 (unstable); aliphatic index = 103.84.

c.515+3G>C: GRAVY = −0.172 (decreased hydrophilicity); instability index = 45.60 (unstable); aliphatic index = 91.39.

c.588+1_588+3delGTA (splicing): GRAVY = −0.126 (decreased hydrophilicity); instability index = 49.84 (unstable); aliphatic index = 89.25.

c.655G>T (p.Glu219*): GRAVY = −0.134 (decreased hydrophilicity); instability index = 50.18 (unstable); aliphatic index = 90.82.

Our results showed that all four unreported novel variants led to a decrease in the GRAVY value, an increase in the aliphatic index, and an elevation in the instability index (II) of the SURF1 protein. These findings suggest a reduction in hydrophilicity, an increase in hydrophobicity, and a decrease in structural stability of the SURF1 protein.

## 4 Discussion

Leigh syndrome (LS), also known as subacute necrotizing encephalopathy, was first reported by Denis Archibald in 1951 (6). This disease has an early onset and is characterized by subacute neurodegenerative lesions as its core feature. Pathogenic variants in the *SURF1* gene are the most common cause of Leigh syndrome, with current estimates suggesting that approximately one-third of Leigh syndrome cases result from *SURF1* deficiency (7). *SURF1* encodes the assembly factor for maintaining the antioxidant of cytochrome c oxidase (COX) stability in the human electron respiratory chain. Mutations in *SURF1* can cause LS, a subacute neurodegenerative encephalopathy, characterized by early onset (infancy), grave prognosis, and predominant symptoms presenting in the basal ganglia, thalamus, brainstem, cerebellum, and peripheral nerves (8, 9). Currently, there is no definitive treatment for *SURF1* deficiency, and only a few patients have been reported to survive beyond age 10 (7). The human *SURF1* gene encodes a 300 amino acid mitochondrial protein necessary for the assembly and maintenance of the COX holoenzyme which is essential for energy production in the human body. The *SURF1* gene is located on chromosome 9p34, in a region of clustering of surfeit genes, where the genomic structure is well-conserved from chicken to human (8).

The SURF1 protein contains three domains, Shy1 domain and two transmembrane, N-terminal and C-terminal domains that play an important role in the function of the protein. Although SURF1 is necessary for the assembly and maintenance of COX, among which the Shy1 domain is involved in the post-translational modification of the mitochondrially encoded Cox1 subunit of cytochrome c oxidase (complex IV), its exact function is not yet well known (10).

The Shy1 domain is an evolutionarily conserved protein domain that was first identified in *Saccharomyces cerevisiae* (baker’s yeast). Its homologous sequences are widely distributed in eukaryotes, including humans, and are closely involved in the assembly of COX. As a core functional region with high evolutionary conservation in theSURF1 protein, the Shy1 domain serves as the key structural basis for SURF1 to exert its mitochondrial functions. Localized in the central region of the protein, this domain is mainly oriented toward the mitochondrial intermembrane space and acts as a core molecular platform for the assembly of complex IV (cytochrome c oxidase, COX). It can interact with COX1 and various assembly factors, thereby regulating the translation, stability, and maturation of COX1, and ensuring the correct formation of the active center of COX. The integrity of the Shy1 domain directly determines whether SURF1 can normally mediate the biosynthesis of complex IV, and it is an essential structure for maintaining mitochondrial respiratory chain function and ensuring cellular energy metabolism (11, 12). Bioinformatics analysis revealed that Shy1 contains a conserved SURF1 domain that links to the biogenesis of complex IV and shares high structural similarity with its homologs in *Saccharomyces cerevisiae* and humans (13). Shy1 is required for the expression of mtDNA-encoded genes and physically interacts with structural subunits and assembly factors of complex IV (14).

**Figure 6:**
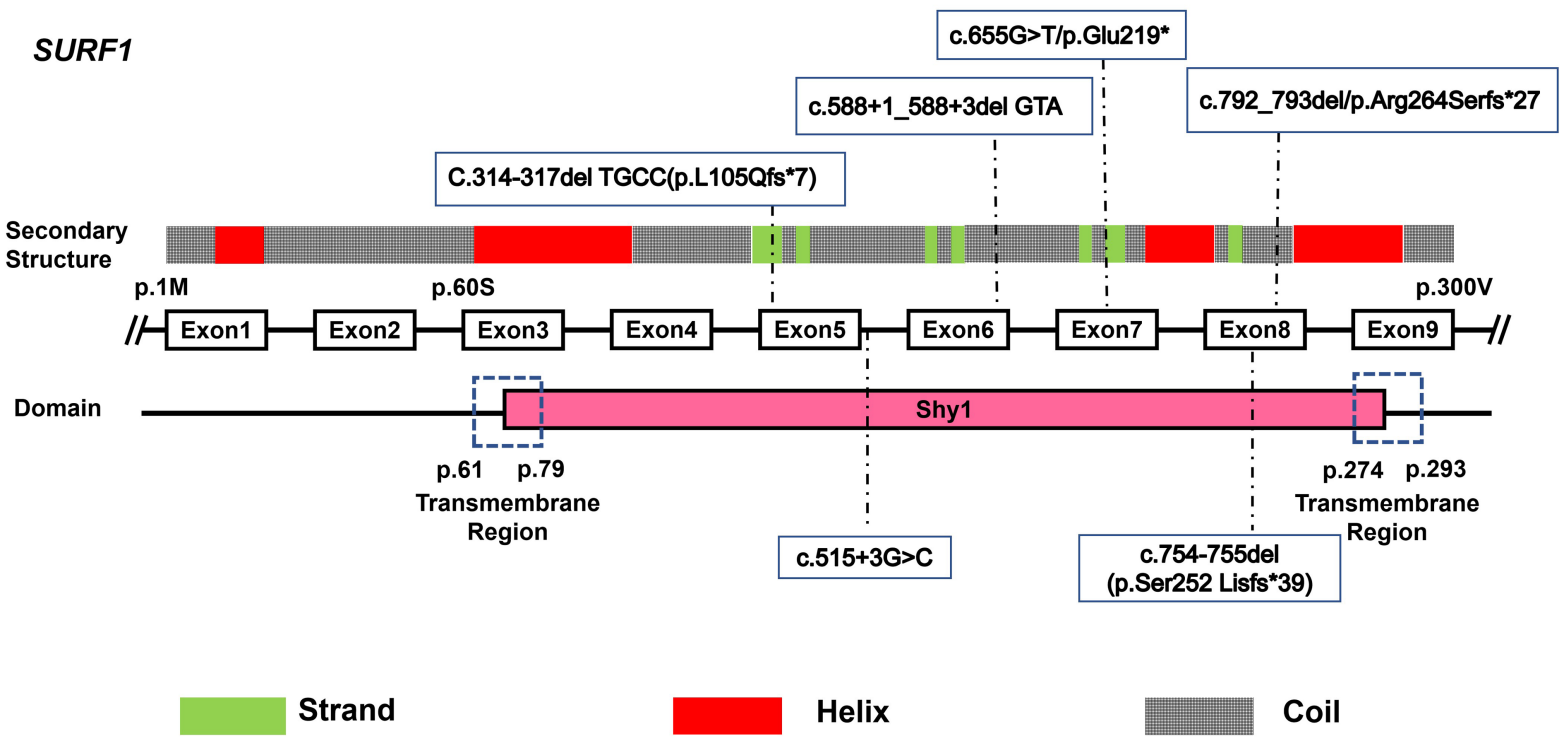
Schematic representation of the SURF1 protein structure with identified variants mapped. The SURF1 protein comprises two transmembrane domains (localized at the N-terminus and C-terminus, respectively) and one evolutionarily conserved Shy1 domain.

Previous studies have confirmed that variants in the N-terminal region, Shy1 domain, or C-terminal region of the SURF1 protein can all lead to unstable COX activity. All six variant sites identified in this study are located in the Shy1 domain, suggesting that they cause abnormal COX activity by altering the post-translational modification of the COX1 subunit, thereby triggering the disease. Secondary structure prediction of the SURF1 protein using I-TASSER software showed that the SURF1 protein is composed of α-helix regions (Helix regions), random coil regions (Coil regions), and β-strand regions (Strand regions). Among these, the Helix and Coil regions are relatively unstable, while the Strand region is relatively stable. Of the six variant sites, five are distributed in the Coil regions, and only c.314-317delTGCC (p.L105Qfs*7) is located in the Strand region (Figure 6).

Leigh syndrome (LS) is a severe neurodegenerative condition with an early onset, typically during early childhood or infancy. The disorder exhibits substantial clinical and genetic diversity. From a clinical standpoint, Leigh syndrome showcases a broad range of irregularities, ranging from severe neurological issues to minimal or no discernible abnormalities. The central nervous system is most affected, resulting in psychomotor retardation, seizures, nystagmus, ophthalmoparesis, optic atrophy, ataxia, dystonia, or respiratory failure. Some patients also experience involvement of the peripheral nervous system, such as polyneuropathy or myopathy, as well as non-neurological anomalies, such as diabetes, short stature, hypertrichosis, cardiomyopathy, anemia, renal failure, vomiting, or diarrhea (15). This can influence cells during development and maybe the reason why developmental delay is the most common feature of LS, as shown in a meta-analysis of the clinical manifestation of LS, where developmental delay was reported as the most common clinical sign in 57% of patients. Developmental delay was followed by respiratory dysfunction (34%), epileptic seizures (33%), poor feeding (29%), and weakness (27%) (16). The majority of SURF1-associated Leigh syndrome cases follow a typical course, leading to early mortality before the age of 10. However, approximately 10% of cases exhibit an atypical progression with milder symptoms and a longer life expectancy (8).

Commonly, resting levels of lactate or pyruvate in the blood are elevated (17). Distinctive observations inpatients with LS typically include the presence of bilateral and symmetrical hyperintensities evident in T2-weighted images. These hyperintensities are primarily located in specific brain regions, notably the basal ganglia (especially the putamen) and various parts of the brainstem, such as the substantia nigra, nucleus ruber, and medulla oblongata (18).

All five children enrolled in this study presented with short stature, developmental delay, hirsutism, and elevated blood lactate levels, which is consistent with the findings of the aforementioned studies. Furthermore, the blood lactate levels of the children were correlated with their clinical symptoms: more severe clinical symptoms, earlier age of onset, and higher blood lactate levels were observed. Brain MRI of four patients showed multiple patchy abnormal signal intensities in the dentate nuclei of the bilateral cerebellar hemispheres, the basis pontis, and the right basal ganglia region, which are consistent with the typical imaging features of LS.

Zhu et al. (19) first confirmed that *SURF1* gene mutations can cause LS in 1998. To date, 814 *SURF1* gene variant sites have been reported, including 23 nonsense mutations, 224 missense mutations, and 73 frameshift mutations. Most of these missense mutations are located in evolutionarily conserved regions, and 90 variant sites can cause LS, which are mainly concentrated in the Shy1 domain (20–24). Nonsense mutations are mainly distributed in exons 4–9, and most are located in the Helix and Coil regions (relatively unstable regions) of the protein secondary structure.

Approximately 11% of human inherited diseases are caused by nonsense mutations, which can generate premature translation termination codons (PTCs) in messenger RNA (mRNA), leading to protein truncation. Nonsense-mediated mRNA decay (NMD) is both a quality control mechanism and a gene regulation pathway. It has been studied for more than 30 years, with an accumulation of many mechanistic details that have often led to debate and hence to different models of NMD activation, particularly in higher eukaryotes (25). NMD is a eukaryotic mRNA surveillance mechanism and regulator of mRNA stability that broadly serves to downregulate premature translation termination codon (PTC)-containing mRNAs. It can degrade transcripts that contain genetic nonsense mutations that occur in ~30% of all human diseases, and/or transcripts resulting from RNA processing errors. Ultimately, NMD can mitigate the harmful effects of such phenomena by limiting the synthesis of the resulting and potentially deleterious C-terminally truncated proteins (26).

Premature translation termination codons (PTCs) usually lead to gene function inactivation and degrade abnormal messenger RNA (mRNA) through the nonsense-mediated mRNA decay (NMD) pathway, resulting in a significant decrease in mRNA levels. This further causes defects in protein synthesis or abundance, leading to various disease phenotypes (27).

The variant sites of Case 1 were c.314-317delTGCC (p.L105Qfs*7) and c.588+1_588+3delGTA (splicing). I-TASSER prediction showed that variations at these two sites caused changes in protein structure. Additionally, wild-type and mutant protein sequences were uploaded to the SWISS-MODEL server, and alignments between the wild-type and mutant proteins were performed using PyMOL software, which revealed differences in protein conformation as shown in the figure. This patient presented with severe clinical symptoms and an early age at death. We speculate that this may be related to the following factors: the variant sites are located in the upstream region of the *SURF1* gene, resulting in a large number of truncated amino acids; the truncated protein includes the deletion of part of the Shy1 domain and the C-terminal region; and the other variant causes abnormal splicing. These dual effects lead to severe impairment of protein function. Due to the family’s refusal to conduct further blood tests, relevant verification experiments were not carried out.

The variant site c.515+3G>C in Case 2 is located in the intronic region between exon 5 and exon 6, which is a novel variant. To verify the pathogenicity of this variant, specific primers were designed for polymerase chain reaction (PCR) detection in this study, and a shortened PCR product was identified. Sequencing analysis confirmed that there was a 192 bp nucleotide deletion in exon 5 of the patient, and the variant product was NM_003172.3: c.324_515del (p.Asp108_Gly172delinsGlu). Specifically, abnormal gene splicing led to the loss of the corresponding amino acids in the translated protein, and this region is located in the Shy1 domain. I-TASSER prediction showed that the amino acids at positions 159, 160, 162, 178, 179, 180, and 181 are located at the ligand-binding sites of the SURF1 protein. However, splicing resulted in the deletion of amino acids 108–172, leading to the partial loss of ligand-binding sites, which prevented effective ligand binding and further affected protein function. Prediction by ProtParam software showed that the spliced protein had decreased hydrophilicity and reduced structural stability.

Protein structure prediction in this study showed that the p.L105Qfs*7, p.Ser252Hisfs*39, p.Arg264Serfs*27, and p.Glu219* variants all resulted in truncation of the amino acid sequence. We uploaded the wild-type and corresponding mutant protein sequences to the SWISS-MODEL server and performed alignments between the wild-type and mutant proteins using PyMOL software, which revealed differences in protein conformation and further verified that the three-dimensional (3D) structure of the protein had changed. Among these variants, p.L105Qfs*7 and p.Glu219* are unreported novel variants. Analysis using ProtParam software confirmed that the corresponding proteins had decreased hydrophilicity, increased hydrophobicity, and reduced structural stability.

RT-qPCR analysis showed that the mRNA expression level of the *SURF1* gene in four patients was significantly lower than that in their parents. Analysis of the ND1/GAPDH ratio revealed that the mtDNA content in three patients was significantly lower than that in their parents and the normal control group (normal children aged 1.5–10 years). We simultaneously verified this using the mtDNA-encoded genesCOX1, COX2, and ND4, and found that the expression levels of COX1, COX2, and ND4 in these three patients were decreased and lower than those in their parents, which further verified that variations at these sites can cause mtDNA depletion.

NMD is a conserved pathway in eukaryotes that quality controls mRNAs and also acts to regulate transcript abundance. mRNAs containing a PTCs are recognized by NMD, which targets them for degradation. Beyond mRNA surveillance, NMD plays an important role in controlling gene expression in mammalian cells, which affects cell cycle regulation, cell viability, response to DNA damage and defense against viral infection. The function of NMD is dual: on the one hand, it can eliminate abnormal transcripts containing PTCs to avoid damage to cells caused by truncated proteins; on the other hand, if the truncated protein still retains partial function, NMD-mediated mRNA degradation may exacerbate the disease phenotype. Dysregulation of the NMD pathway has been implicated in severe pathologies, including neuro-developmental disorders, cellular stress, and cancer (28, 29).

Understanding the molecular mechanisms of NMD is therefore of paramount importance for the development of new treatment strategies aimed at modulating the function and activity of the proteins involved in NMD. Previous studies have confirmed that caffeine and Wortmannin can inhibit the activity of SMG1 kinase, 5-azacytidine can promote the expression of c-Myc, cardiac glycosides (such as digoxin and ouabain) can increase cytoplasmic calcium concentration, and Upf3b-ASO can stabilize mRNA in mouse models of muscular dystrophy and hemophilia IX deficiency. All the aforementioned substances can inhibit the NMD pathway (28, 30). Duchenne muscular dystrophy (DMD) is a muscle-degenerating disease caused by mutations in the DMD gene. Researchers used splice-switching antisense oligonucleotides (ASOs) to induce the skipping of out-of-frame exons of DMD, aiming to introduce PTCs (31). Therefore, pharmacological modulation of the NMD pathway may represent a potential therapeutic strategy for nonsense mutation-associated LS.

## 5 Conclusion

This study reported the clinical and gene characteristics of a cohort of Chinese pediatric patients with Leigh syndrome (LS) associated with *SURF1* gene variants. Four novel unreported variants were identified: c.314-317delTGCC (p.L105Qfs*7), c.588+1_588+3delGTA (splicing), c.655G>T (p.Glu219*), and c.515+3G>C. Through experimental assays, bioinformatic analyses, and literature review, the pathogenicity and underlying mechanisms of these distinct variants were investigated. *SURF1* gene variants lead to amino acid alterations, resulting in changed protein structure, decreased hydrophilicity, increased hydrophobicity, and reduced stability. These variants may also trigger mRNA degradation and mitochondrial DNA depletion, ultimately leading to abnormal COX activity and the development of LS.

As a rare disease, LS relies on genetic testing not only as an important diagnostic tool but also as a reference for the formulation of subsequent therapeutic strategies. In this study, we found that different variant sites corresponded to distinct clinical phenotypes. Patients 3 and 4 carried the same variant but exhibited slight differences in clinical presentation. We speculate that this may be related to family environment, early identification of the variant and early rehabilitation intervention, as well as the young age of the patients and atypical clinical manifestations. We are unable to conduct additional clinical trials for further validation due to limited conditions.

However, the present study has several limitations. For instance, the sample size was relatively small, functional studies *in vitro* were lacking, and modifier gene screening was not performed, we are unable to perform the site-specific mechanistic analysis and in-depth exploration. In the future, we aim to expand the cohort of Chinese children, establish cell or animal models with SURF1 mutations, and explore targeted therapies for COX deficiency.

## Data Availability

The original contributions presented in the study are included in the article/supplementary material, further inquiries can be directed to the corresponding author.

## Footnotes

1
https://web.expasy.org/protscale/

2
https://web.expasy.org/protparam/
